# Developing a Brief Cognitive Task Intervention to Reduce Long-Standing Intrusive Memories of Trauma: A Feasibility Study With Remote Delivery for Women in Iceland

**DOI:** 10.32872/cpe.11237

**Published:** 2024-03-28

**Authors:** Johann Palmar Hardarson, Beau Gamble, Kristjana Thorarinsdottir, Elín Sjöfn Stephensen, Marie Kanstrup, Thorsteinn Gudmundsson, Unnur Valdimarsdóttir, Arna Hauksdottir, Andri S. Bjornsson, Michelle L. Moulds, Emily A. Holmes

**Affiliations:** 1 Department of Psychology, University of Iceland, Reykjavik, Iceland; 2 Department of Psychology, Uppsala University, Uppsala, Sweden; 3 The Center of Public Health Sciences, University of Iceland, Reykjavik, Iceland; 4 Department of Epidemiology, Harvard TH Chan School of Public Health, Boston, MA, USA; 5 The National University Hospital of Iceland, University of Iceland, Reykjavik, Iceland; 6 School of Psychology, UNSW Sydney, Sydney, Australia; 7 Department of Women’s and Children’s Health, Uppsala University, Uppsala, Sweden; Philipps-University of Marburg, Marburg, Germany

**Keywords:** trauma, intrusive memories, intervention, feasibility study, mental imagery

## Abstract

**Background:**

There is emerging evidence that a brief cognitive task intervention may reduce the frequency of intrusive memories, even long-standing memories of older trauma. However, evaluations to date have involved in-person researcher contact. We investigated the feasibility and acceptability of remote delivery to women (n = 12) in Iceland who had experienced trauma on average two decades earlier.

**Method:**

Participants monitored intrusive memories in a daily diary for one week (i.e., baseline phase), completed (at least) two guided, remote intervention sessions (e.g., via secure video platform), and were encouraged to continue to use the intervention self-guided.

**Results:**

Eight participants completed the primary outcome and reported fewer intrusive memories in Week 5 (M = 6.98, SD = 5.73) compared to baseline (M = 25.98, SD = 29.39) – a 68% reduction. Intrusions decreased at each subsequent time point; at 3-months (n = 7) there was a 91% reduction compared to baseline. Other psychological symptoms reduced and functioning improved. Importantly, participant ratings and qualitative feedback support feasibility and acceptability.

**Conclusion:**

Findings suggest the feasibility of remote delivery of the brief imagery-competing task intervention by non-specialists (who were not mental health professionals) and hold promise for developing psychotherapeutic innovations supporting women with intrusive memories even decades after trauma.

## Highlights

- There is a high global prevalence of trauma exposure and mental health resources are limited.
- The intervention delivered remotely by non-specialists to women in Iceland was feasible and acceptable.
- Participants reported fewer intrusive memories at 5 weeks post-intervention relative to the baseline phase.
- This method could complement existing therapies, in cases of long waitlists or lack of access.

Effective brief, low intensity interventions are needed to address mental health problems on a global scale. Such an intervention has been developed to target intrusive trauma memories (Holmes et al., 2009; Iyadurai et al., 2018; Kanstrup, Singh, et al., 2021). The intervention draws on cognitive neuroscience (Monfils & Holmes, 2018), specifically targeting the potential effect of taxing working memory on altering re-consolidation of trauma memories (Visser et al., 2018). It comprises three components: (1) briefly bringing a trauma memory to mind, (2) engaging in a visuospatial task such as the computer game ‘Tetris’ for approximately 20 minutes, whilst (3) employing mental rotation during gameplay. Studies in the laboratory (using trauma analogues; e.g., James et al., 2015) and with trauma exposed samples (e.g., women who experienced traumatic childbirth, Horsch et al., 2017; emergency department patients, Iyadurai et al., 2018; Kanstrup, Singh, et al., 2021) demonstrate that receiving the intervention in the initial hours and days posttrauma results in fewer intrusive memories relative to receiving a placebo control.

There is also emerging evidence that this intervention reduces long-standing intrusive memories up to decades old; e.g., in people with chronic PTSD (Kanstrup, Kontio, et al., 2021; Kessler et al., 2018). Further, a pilot case study (Thorarinsdottir et al., 2021) and brief case series (*N* = 3; Thorarinsdottir et al., 2022) with Icelandic women with a chronic trauma history provided preliminary evidence of its capacity to reduce intrusive memories in this group. Not only were treatment gains (i.e., reduced intrusions) maintained at 3 month follow-up, other clinical symptoms (e.g., depression, anxiety) reduced and functioning (e.g., concentration, sleep) improved.

Essential to an intervention’s scope for scalability is its capacity for effective remote delivery, eliminating the need for in-person contact. Ideally, scalable interventions should be deliverable by non-specialists who have received remotely-delivered training. Whilst the abovementioned case study (Thorarinsdottir et al., 2021) and case series (Thorarinsdottir et al., 2022) provide encouraging preliminary evidence of the cognitive task intervention’s effectiveness, both studies included some aspects of in-person recruitment and/or intervention delivery, and the intervention was delivered by a qualified clinical psychologist.

In line with the goal of establishing scalability, the current study (i) investigated the feasibility of a fully remote delivered, researcher-guided form of the intervention, and (ii) explored pre- to post-intervention changes in the number of intrusive memories. In addition, we delivered some aspects of the intervention in digitalized format; i.e., via brief animated film-clips (e.g., to explain the target symptom).

We investigated feasibility in a sample of trauma-exposed women in Iceland who reported intrusive memories of long-standing trauma. We assessed the feasibility of delivering the intervention in a fully remote format based on the number of sessions completed, dropout rates and reasons, and adverse events. We also investigated the feasibility of conducting remote training and supervision of non-specialists (psychology students) to train them to deliver the intervention. Finally, we assessed intervention acceptability via participants’ ratings and qualitative feedback.

Consistent with previous studies (e.g., Kanstrup, Singh, et al., 2021), we predicted that, compared to the baseline phase (Week -1), participants would report fewer intrusive memories in the fifth week after the second intervention session, as assessed via a daily diary (primary outcome). Second, we predicted that the intervention would lead to reductions in related psychological symptoms (e.g., anxiety, depression), and improved functioning (e.g., in concentration, sleep, social relationships) (secondary outcomes). We also aimed to explore whether the frequency of targeted intrusive memories decreased relative to the frequency of non-targeted intrusive memories.

## Method

### Participants

Women in a sub-study of the Stress and Gene Analysis (SAGA) cohort (a population-based longitudinal study of Icelandic women investigating trauma history, www.áfallasaga.is) were screened for eligibility. The sub-study (the Social Trauma Project) involves a comparative analysis of two sub-samples extracted from the SAGA cohort (i.e., women with likely PTSD or no PTSD). Participants were assessed (in person) with the Clinician-Administered PTSD Scale for DSM-5 (CAPS-5; Weathers et al., 2018) and the Mini International Neuropsychiatric Interview for DSM-4 (MINI; Sheehan et al., 1998). These diagnostic interviews were adminstered by fully qualified clinical psychologists and students who were completing their Masters in Clinical Psychology.

Inclusion criteria were: (a) having experienced at least one Criterion A trauma according to the Diagnostic and Statistical Manual of Mental Disorders (5th Ed.; DSM–5; American Psychiatric Association, 2013); (b) reporting at least two intrusive memories in the previous week (consistent with the criterion of a minimum of 1-2 intrusions per week required to endorse this symptom on the CAPS-5); (c) reporting being bothered by intrusive memories over the past month (i.e., scoring at least a moderate score on PCL-5 item 1); (d) able and willing to complete 3-9 sessions with the researcher; (e) willing to monitor intrusive memories; (f) having access to a smartphone; (g) able to speak Icelandic and read study materials in Icelandic. Exclusion criteria (assessed with the MINI) were: (a) current psychotic disorder; (b) current manic episode; (c) being acutely suicidal.

Twelve women were enrolled in the study (mean age = 42.42 years, *SD* = 12.03; mean duration since time of trauma (target memory) = 20.73 years, *SD* = 14.65). Primary traumas were sexual violence (*n* = 5), witness to death or serious injury (*n* = 3), physical violence (*n* = 3), and motor vehicle accident (*n* = 1). Eight participants completed the intervention and the primary outcome; 7 participants completed the 3-month follow-up.

### Design

Participants monitored intrusive memories of trauma in a daily diary for one week (i.e., baseline phase, Week -1), followed by at least two guided intervention sessions with a researcher remotely (via telephone or secure video platform) over the following week (Week 0). Participants could opt to complete up to four additional guided intervention sessions (i.e., maximum of 6 sessions), until the completion of the primary outcome (i.e., Week 5). After the first guided session, participants were encouraged to use the intervention on their own throughout the study.

Participants continued to monitor their intrusive memories in the daily diary throughout Weeks 0-5. Follow-up questionnaires were completed at Week 1, 1-month, and 3-months after the second intervention session. The primary outcome was the change in total number of intrusive memories from the baseline week (Week -1) to the fifth week after the second intervention session (Week 5). Participants also monitored (in a daily diary) the number of intrusive memories they experienced for one week, beginning the day of completing the 3-month follow-up questionnaires.

The study had a *repeated AB design*, such that the length of baseline (‘A,’ preintervention, monitoring only) and intervention (‘B’) phases differed across each intrusive memory; i.e., depending on when it was targeted. The baseline phase could thus be used as a control period for each individual memory – i.e., to compare the number of intrusive memories before and after the intervention.

### Training and Supervision of Psychology Students to Deliver the Intervention

The intervention was delivered to the first participant by KT, a licensed clinical psychologist who had received training in delivering the intervention via two workshops led by EAH and MK, and had experience in intervention delivery (Thorarinsdottir et al., 2021). The intervention was delivered to the next 11 participants by four MSc students in clinical psychology and two BSc students at the University of Iceland who received remotely-delivered training and ongoing supervision.

To allow remote training during the COVID-19 pandemic, we developed a beta version of an online training course (via the platform www.talentlms.com) in the style of a ‘MOOC’ (massive open online course), which included material in the form of text, images, animated videos, video roleplay assignments, quizzes, and written reflections (Oakley & Sejnowski, 2019). Alongside the MOOC, training was delivered by KT, JPH, and MK (all with intervention delivery experience [Kanstrup, Kontio, et al., 2021; Kanstrup, Singh, et al., 2021; Thorarinsdottir et al., 2021, 2022]) and supervised by ASB and EAH.

A training group (trainees, trainers, facilitator (BG) and supervisors) met via Zoom for seven one-hour weekly sessions (Sept-Nov 2020), as trainees worked through the online course. Trainees could discuss the MOOC, observe experienced trainers roleplaying and ask questions. Trainees uploaded video roleplays online, assessed by KT and JPH with rating scales ranging from 0 (‘absence’) to 6 (‘excellence’) covering nine components (e.g., *‘Explanation of the target symptom (intrusive memories)*). Trainees were required to score at least 4 (‘competent’) on all scales before delivering the intervention.

The group continued to attend weekly Zoom supervision meetings (Jan-July 2021) with the option of individual supervision (from KT).

### Measures

#### Primary Outcome Measure

*Intrusive memory diary.* Participants monitored their intrusive memories in a daily paper diary used in previous research (e.g., Iyadurai et al., 2018; Kanstrup, Singh, et al., 2021) and validated (Singh, Ahmed Pihlgren, et al., 2023). Primary outcome was the change in total number of intrusive memories of the traumatic event recorded in the diary (morning, afternoon, evening and night) from baseline week (Week -1) to the fifth week after the second intervention session (Week 5).

#### Secondary Outcome Measures

In line with the goal of investigating feasibility and in the interest of brevity, we report data for the first five pre-registered ‘Secondary Outcome measures’ which examine symptoms of PTSD, depression, and anxiety along with intrusive memories. Findings for the remaining measures including functional measures are presented in Appendix A of the Supplementary Materials.

*Intrusive memory diary.* Change in the total number of intrusive memories recorded in the diary daily during the week of receiving the first two intervention sessions (Week 0), the subsequent four weeks (Weeks 1-4) and at 3-month follow-up, compared to the Baseline week (Week -1).

*Unwanted Memories of Trauma* (*UMT*; Hackmann et al., 2004). Six items measuring the frequency of unwanted memories of the trauma in the previous week, the level of distress, nowness, reliving, disconnectedness associated with intrusions, and the degree to which different triggers are associated with memories of the trauma.

*Posttraumatic Stress Disorder Checklist 5 (PCL-5*; Weathers et al., 2013). A 20-item measure assessing the severity of PTSD symptoms.

*Patient Health Questionnaire-9 (PHQ-9;* Kroenke et al., 2001). A 9-item measure of the severity of depression symptoms.

*Generalized Anxiety Disorder-7* (*GAD-7*; Spitzer et al., 2006). A 7-item screening tool assessing the presence and severity of GAD symptoms.

*Sheehan Disability Scale* (*SDS*; Leon et al., 1997). A measure of functional impairment in work/school, social and family life domains. Items were adapted to assess functional impairment associated with intrusive memories.

*World Health Organization Disability Assessment Schedule 2.0 (WHODAS 2.0*; World Health Organization, 2010). A 12-item questionnaire measuring difficulties due to health conditions, including mental problems. Lower scores indicate better functioning.

*Impact of intrusive memories on concentration, sleep and stress – Ratings.* Self-rated items assessing the impact of intrusive memories on concentration, sleep and stress in the past week. Two items assess general concentration difficulties and impairments in concentration, two items assess sleep disturbances, and one item assesses the impact of intrusive memories on stress.

*Rating of how long intrusive memories disrupt concentration.* A single item assessing the estimated average duration of disruption to concentration, rated on a 6-point scale (from 0 = *<1 minute* to 6 = *> 60 minutes*).

*Impact of intrusive memories on functioning*. A 2-item measure assessing the impact of intrusive memories on daily functioning. The first question is: "*Have the intrusive memories affected your ability to function in your daily life in the past week?* (from 0 = *not at all* to 10 = *affected very much*), followed by the open-ended question: *"If yes, how?"*.

*General impact of intrusive memories – Ratings*. Two items assessing the impact of intrusive memories.

#### Other Outcome Measures

Only data for the pre-registered ‘Other Outcome Measures’ that examine feasibility, adherence, and acceptability are reported and described, in the interest of conciseness. For a comprehensive review of the remaining Other Outcome Measures, please refer to the CTR (NCT04709822); the corresponding findings can be found in Appendix B of the Supplementary Materials.

*Self-guided intervention adherence – Usage of the gameplay intervention in daily life.* Two items assessing participants’ use of the gameplay intervention in everyday life: *"How many times did you manage to play Tetris after you experienced an intrusive memory?"* If relevant participants were asked a follow-up open-ended question, i.e.: "*Which of your intrusive memories did you target when you played on your own?"*.

*Intrusion diary adherence.* A single item assessing participants’ adherence to completing the intrusion diary accurately.

*Acceptability ratings*. Acceptability of the intervention was assessed with two rating items. Acceptability was also assessed with two open-ended questions ("*How did you feel about playing Tetris after you had an intrusive memory?"* and *"Did you find the intervention helpful? If yes, how?").*

*Credibility/expectancy scale*. Prior to completing the intervention for the first time, participants provided ratings of treatment expectancy^1^1The CTR states that this scale contained 5 items, but only 4 items were included owing to an administrative error. as well as the degree to which they found the rationale for intervention credible. Wording of the items was adapted for the current study.

### Procedure

Participants were recruited between January and May 2021. Women who participated in the Social Trauma Project sub-study of the SAGA cohort who met the inclusion criteria were contacted (described in Thorarinsdottir et al., 2021).

#### Baseline Session

Eligible participants were invited to a remote meeting (i.e., baseline session) with a researcher. Participants were given a brief verbal description of the study, presented with an information sheet containing study details, and provided informed consent by signing an e-consent form in the electronic registration system REDCap. All but one participant indicated that they had a printer and were emailed the diary. A paper diary was delivered to the remaining participant.

Participants then watched a brief video titled *What are intrusive memories?* The researcher asked a series of questions to check their understanding of the content, then sent a link to a second video, *Identifying your intrusive memories.* Participants were then asked to generate a list of intrusive memories they were experiencing. The researcher emphasised that they should not provide a detailed description of each intrusion, but rather summarise each briefly in only a few words (e.g., “*dark room*”). The researcher recorded each intrusive memory in REDCap and shared the screen containing the list of intrusive memories with each participant.

Next, participants watched the third video, *Keeping count of your intrusive memories,* which explained how to monitor intrusive memories. The researcher then explained how to use the intrusive memory diary to monitor their daily intrusions in the week ahead (i.e., baseline, Week -1). Participants also completed baseline questionnaires, and an appointment was scheduled for the first intervention session.

#### First Intervention Session

At the start of the session, the researcher explained that the session would involve using the intervention to target one of the participant’s intrusive memories, then sent them a link to the fourth video (*What is the intervention?*) which provided a rationale.

Together the participant and researcher then chose an intrusion to target (typically the most frequent or distressing). Next, the researcher asked participants to briefly bring the memory to mind so they could ‘see it in their mind’s eye’, but without discussing its content. The researcher sent a link to a fifth video (*How to play Tetris using mental rotation*), which included instructions about how to play Tetris, and emphasised the importance of mental rotation (i.e., mentally rotating upcoming blocks in the game to visualise how to best place them). After viewing, the researcher directed participants to open www.tetris.com in their web browser and share their screen with the researcher. Participants then had the opportunity to practice playing Tetris (if they wanted to) and were then instructed to engage in gameplay using mental rotation for at least 20 minutes. Next, an appointment was scheduled for the second intervention session, and participants were also given instructions as to how to use the intervention at home. The last three participants also watched a final video, *Tetris and the brain,* which re-iterated the rationale for the intervention and its hypothesised mechanisms.

#### Second Intervention Session

Participants received a second intervention session approximately one week later, targeting the same intrusion (i.e., if intrusions persisted) or a different intrusive memory that they wished to reduce. Participants were informed that they had the choice of continuing to use the intervention alone (i.e., self-guided) or scheduling further intervention session/s (up to 6 sessions) with researcher support.

Participants continued to monitor the frequency of both targeted and non-targeted intrusive memories in the daily diary throughout Weeks 0-5.

#### Follow-Up Assessments

Participants completed follow-up questionnaires at Week 1, 1-month, and 3-months after the second intervention session. Participants also monitored their intrusive memories for one week at the 3-month follow-up.

At each intervention session and assessment, participants were asked about the occurrence of any adverse events since the previous contact.

This study was registered on ClinicalTrials.gov (NCT04709822) on 14/1/2021. It was approved by the National Bioethics Committee in Iceland (ID: No. VSNb2017110046/03.01, dated 1/10/2019; amendments: (i) 17-238-V23, dated 23/6/2020; (ii) 17-238-V27, dated 24/11/2020; (iii) 17-238-V29-S1, dated 2/3/2021; (iv) 17-238-V30, dated 30/3/2021; (v) 17-238-V31, dated 13/4/2021). Participants provided their informed consent digitally. All sessions followed a written protocol. No serious adverse events or adverse events related to the intervention were reported.

## Results

### Analytic Approach

As a feasibility trial, we adopted a descriptive approach to reporting the results. Whilst we collected both qualitative and quantitative data, only quantitative findings are reported here. Analyses were conducted (by BG) using R, Version 4.0.242 (‘psych’ package, version 2.0.8, for descriptive analyses). De-identified summary data, codebook and R scripts are available on the Open Science Framework (Gamble et al., 2022). Whilst descriptive statistics for all participants are reported below, we also provide data for completers only (i.e., per protocol analyses) on the OSF (Gamble et al., 2022).

**Figure 1:**
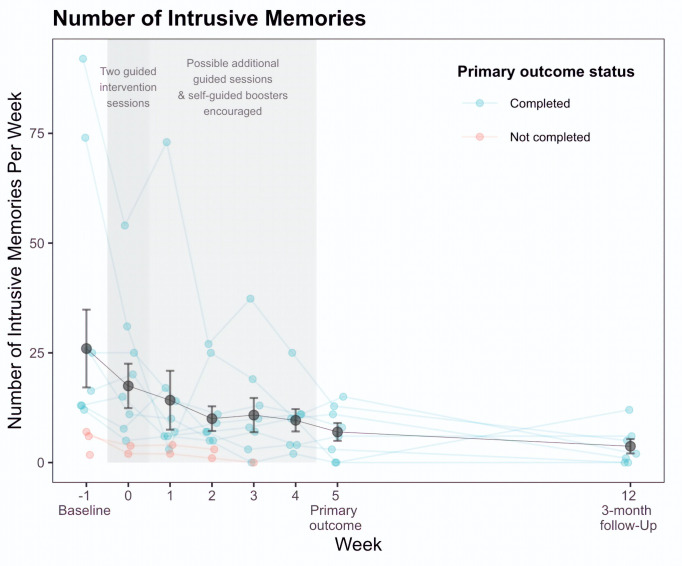
Number of Intrusive Memories for All Participants (*n* = 12): Treatment Completers (*n* = 8) and Non-Completers (*n* = 4)

Figure 1 presents the number of intrusive memories reported in the daily diary for each participant. Table 1 reports the means, *SD*s, and effect sizes (as Cohen’s *d* along with 95% CIs) for (i) number of intrusive memories reported in the daily diary at each assessment point, and (ii) secondary outcome measures at each assessment point. Table 2 reports measures of adherence and credibility.

**Table 1:** Number of Intrusive Memories Reported in the Daily Diary and Self-Report Measures of Posttraumatic Stress Symptoms, Depression and Anxiety for All Participants (n = 12)

Outcome	*n*	*M*	*SD*	Cohen’s *d* Comparison to baseline	Cohen’s *d* 95% CI	
					*LL*	*UL*
Number of intrusive memories (daily diary)						
Baseline (Week -1)	11	25.98	29.39			
Week 0	10	17.46	16.01	-0.27	-0.55	0.00
Week 1	10	14.20	21.21	-0.52	-1.05	0.02
Week 2	10	10.00	8.91	-0.22	-0.39	-0.05
Week 3	9	10.81	11.69	-0.38	-0.66	0.10
Week 4	8	9.62	7.19	-0.64	-1.22	-0.07
Week 5	8	6.98	5.73	-1.16	-2.47	0.15
3-month	7	3.71	4.35	-0.81	-1.52	-0.11
UMT^a^ (frequency)						
Baseline	12	3.25	1.14			
Week 1	9	3.56	1.33	0.09	-0.71	0.89
1-month	8	2.38	0.74	-1.52	-2.98	-0.06
3-month	7	1.29	0.76	-2.36	-3.59	-1.14
UMT^a^ (distress)						
Baseline	12	45.42	15.08			
Week 1	9	41.33	19.40	-0.28	-0.79	0.24
1-month	8	28.50	22.58	-0.94	-1.69	-0.18
3-month	7	24.00	29.45	-0.76	-1.52	0.01
UMT^a^ (nowness)						
Baseline	12	38.83	25.46			
Week 1	9	25.56	22.56	-0.63	-1.50	0.25
1-month	8	23.00	23.86	-0.77	-1.75	0.22
3-month	7	10.43	26.72	-1.10	-2.32	0.12
UMT^a^ (reliving)						
Baseline	12	40.58	24.83			
Week 1	9	39.22	29.53	-0.20	-1.12	0.73
1-month	8	30.38	29.40	-0.36	-1.40	0.69
3-month	7	17.71	26.02	-0.83	-1.91	0.26
UMT^a^ (disconnectedness)						
Baseline	12	61.42	24.99			
Week 1	9	72.78	17.37	0.38	-0.60	1.35
1-month	8	59.88	17.11	-0.03	-0.97	0.92
3-month	7	32.14	37.81	-0.81	-2.30	0.68
UMT^a^ (triggers)						
Baseline	12	55.67	24.63			
Week 1	9	52.89	28.87	-0.36	-1.04	0.32
1-month	8	35.88	27.76	-0.65	-1.29	-0.01
3-month	7	28.71	28.62	-0.79	-1.77	0.18
PCL-5^b^						
Baseline	12	36.42	16.81			
Week 1	9	28.89	16.83	-0.42	-0.77	-0.07
1-month	8	28.50	21.27	-0.31	-0.73	0.10
3-month	7	18.71	17.53	-0.71	-1.37	-0.05
PHQ-9^c^						
Baseline	12	11.83	5.70			
Week 1	9	9.89	4.76	-0.20	-0.53	0.13
1-month	8	8.62	3.46	-0.37	-0.71	-0.02
3-month	7	8.29	5.71	-0.46	-0.98	0.07
GAD-7^d^						
Baseline	12	8.75	6.17			
Week 1	9	6.00	4.39	-0.16	-0.34	0.02
1-month	8	5.62	4.00	-0.30	-0.57	-0.03
3-month	7	5.00	4.62	-0.44	-1.18	0.31

^a^Unwanted Memories of Trauma. ^b^Posttraumatic Stress Disorder Checklist 5. ^c^Patient Health Questionnaire-9. ^d^Generalized Anxiety Disorder-7.

**Table 2:** Self-Report Measures of Ratings of Adherence and Credibility/Expectancy for All Participants (n = 12)

Outcome	*n*	*M*	*SD*
Self-Guided Intervention Adherence			
Baseline			
Week 1	9	3.67	3.20
1-month	8	4.00	3.38
3-month	7	2.86	2.34
Intrusive Memory Diary Adherence			
Baseline	11	8.27	1.01
Week 0	10	7.70	1.42
Week 1	10	7.90	1.10
Week 2	10	8.00	1.15
Week 3	9	7.44	1.33
Week 4	8	8.06	1.02
Week 5	8	7.94	1.21
Week 12	6	8.67	1.37
Credibility/Expectancy – How logical			
Baseline	12	74.67	20.03
Credibility/Expectancy – How useful			
Baseline	12	75.42	18.42
Credibility/Expectancy – How strongly recommend to a friend			
Baseline	12	65.50	17.96
Credibility/Expectancy – How much improvement expected			
Baseline	12	69.67	16.52

### Primary Outcome

The primary outcome was the change in the total number of intrusive memories recorded in the daily diary from baseline (Week -1) to Week 5. Participants reported fewer intrusive memories of the traumatic event in the fifth week after the second intervention session (*M =* 6.98, *SD* = 5.73, range: 0-15) compared to the baseline week (Week -1; *M* = 25.98, *SD* = 29.39, range: 2-92) – a difference that reflected a 68% reduction in the number of intrusions (i.e., for participants who completed the primary outcome, *n* = 8).

### Secondary Outcomes

We explored whether participants reported fewer intrusive memories in the daily diary at Week 0, Weeks 1-4 and at 3-month follow-up (relative to Week -1, baseline phase), as well as reductions in other psychological symptoms (e.g., anxiety, depression) over the course of the study (see Table 1 for means).

#### Change in Total Number of Intrusive Memories

Participants recorded fewer intrusive memories in the diary during the week of receiving the first two intervention sessions (Week 0), the subsequent four weeks (Weeks 1-4) and at 3-month follow-up, relative to the baseline week (i.e., Week -1). At 3-month follow-up, there was a 91% reduction in the number of intrusive memories reported relative to baseline (Week -1) (i.e., for participants who completed the 3-month follow-up, *n* = 7).

#### Symptoms of PTSD

*Unwanted Memories of Trauma (UMT).* Overall, participants reported increased frequency of intrusive memories from baseline to Week 1; however, ratings of frequency declined across subsequent time points. Similarly, ratings of disconnectedness increased from baseline to Week 1, but progressively diminished at each subsequent time point. For the remaining items (distress, nowness, reliving, triggers), participants’ ratings steadily decreased from baseline to 3-month follow-up.

*Posttraumatic Stress Disorder Checklist 5 (PCL-5).* PCL-5 scores decreased at each time point, from baseline to 3-month follow-up.

#### Depression and Anxiety Symptoms

*Patient Health Questionnaire-9 (PHQ-9)*. PHQ-9 scores decreased at each successive time point, from baseline to 3-month follow-up.

*Generalized Anxiety Disorder-7 scale (GAD-7)*. Anxiety symptoms decreased at each assessment point, from baseline to 3-month follow-up.

#### Functioning

*Sheehan Disability Scale (SDS)*. Ratings of functional impairment decreased at each assessment point for all domains, indicating improved self-reported functioning from baseline to 3-month follow-up.

*World Health Organization Disability Assessment Schedule 2.0 (WHODAS 2.0)*. Consistent with the SDS, participants reported improvements in functioning at each timepoint, across the course of the study.

*Impact of intrusive memories on concentration, sleep, and stress – Ratings*. Participants reported improved concentration at each timepoint. Ratings of the impact of intrusive memories on sleep decreased at each timepoint from baseline to 1-month follow-up. However, participants rated an increased impact of their intrusions on sleep from the 1-month to 3-month follow-up. Notably, the mean rating at 3-month follow-up was lower than that reported at baseline. Regarding nightmares, ratings indicated an increased impact of intrusions on nightmares from baseline to Week 1, with decreased ratings at each subsequent timepoint (with a mean of 0 at 3-month follow-up). Finally, although participants reported an overall reduction in the impact of intrusive memories on stress across the study, the means fluctuated across assessment points. Specifically, ratings reduced (indicating that intrusions had less impact on stress) from baseline to Week 1, then increased at 1-month follow-up, and subsequently decreased at 3-month follow-up.

*Rating of how long intrusive memories disrupted concentration on average*. Ratings indicated that the duration of time that intrusive memories disrupted concentration decreased from baseline to 3-month follow-up. Whilst duration of disruption increased from baseline to 1 week, it decreased at each subsequent timepoint.

*Impact of intrusive memories on functioning*. Ratings of the impact of intrusive memories decreased at each assessment point, from baseline to 3-month follow-up.

*General impact of intrusive memories – Ratings.* Ratings of the vividness of intrusive memories and intrusion-related distress reduced from baseline to 3-month follow-up. Despite these overall reductions there was some fluctuation across assessment points.

We also planned to explore the relative differences in the number of intrusive memories (reported during the baseline phase and Week -1) targeted by the intervention and non-targeted intrusive memories. However, we were unable to conduct these planned exploratory analyses because participants’ untargeted intrusive memories were not sufficiently frequent to conduct the comparisons. Whilst such analyses have been carried out in a previous investigation (Kessler et al., 2018), we note that participants in that study were inpatients with complex PTSD who reported frequent intrusive memories of multiple traumas. By comparison, in the current feasibility trial participants reported a smaller number of key intrusive memories, which were the focus of the intervention – and non-targeted intrusions were less frequent.

#### Feasibility

*Feasibility of delivering the intervention in fully-remote format.* Twelve participants commenced the trial, of whom 8 completed the primary outcome. Seven treatment completers completed the required two intervention sessions, and one completed four sessions. Of the four non-completers, two completed two intervention sessions, one completed one session, and one completed zero sessions. Two completed the Week 1 follow-up but could not be contacted to obtain the primary outcome. The other two dropped out before the Week 1 follow-up due to unrelated stressors. No adverse events were reported.

*Feasibility of remote training and supervision to deliver the intervention.* All four MSc students and the two BSc students attended all remote training sessions and completed the online training course. Online supervision sessions proved feasible, enabling interactions between the students and trainers in real-time, and the opportunity for practical teaching components such as role-plays.

*Feasibility of training non-specialists (i.e., BSc and MSc students) to deliver the intervention to a competent standard.* All trainees were judged to reach competence (defined as scoring a ‘4’ or greater on all competency rating scales), demonstrating the feasibility of training non-specialists to deliver the intervention. The median competency score across trainees for all rating scales was 5.00, based on the final round of video roleplays completed prior to delivering the intervention to participants.

#### Adherence

Participants’ ratings of self-guided intervention adherence (i.e., usage in everyday life) indicated that (across all time points) the average number of times participants played Tetris after experiencing an intrusive memory was 3.54 (*SD* = 2.95). These ratings were relatively consistent from Week 1 to 3-month follow-up (range = 2.86 – 4.00; see Table 2). There were high levels of adherence to completing the daily diary: of the 8 participants who completed the primary outcome, the mean percentage of missing days (across all weeks) was 2.27% (*SD =* 4.02%). In addition, participants’ self-rated accuracy in completing the diary indicated consistently high levels of accuracy (*M* = 7.96, *SD* = 1.19) across all time points.

#### Acceptability

Participants’ ratings indicated acceptability. Specifically, participants indicated that they would recommend the intervention to a friend (*M =* 6.50, *SD* = 3.51), and considered gameplay an acceptable way to reduce intrusive memories (*M =* 6.25, *SD* = 3.20).

#### Credibility/Expectancy

Overall, participants rated high levels of intervention expectancy and credibility (*M* = 71.31, *SD* = 14.43).

## Discussion

We investigated the feasibility and acceptability of a remotely delivered, researcher-guided imagery-competing task intervention targeting intrusive memories of long-standing trauma in a sample of women in Iceland. Twelve participants commenced the trial, of whom 8 completed the primary outcome. No intervention related adverse events were reported. These data confirm the feasibility of remote delivery of the researcher-guided form of the intervention and good client engagement. The trial also confirmed the feasibility of conducting remote (i.e., fully online) training for non-specialists without clinical psychology qualifications. Finally, participants’ ratings indicated acceptability of the remote version of this brief guided intervention.

Another goal was to explore pre- to post-intervention changes in the number of intrusive memories reported in a daily diary. As predicted, participants reported fewer intrusive memories in Week 5 relative to baseline; specifically, a 68% reduction. By 3-month follow-up, there was a 91% reduction relative to baseline. This pattern of improvement was also observed across other psychological outcomes: depression and anxiety symptoms reduced, and self-reported functioning improved. These encouraging results extend the findings of our previous case series’ using the same imagery competing task intervention, in which participants (*n* = 1, *n* = 3) reported a reduced frequency of intrusive memories by 38% to 56% in the intervention phase, with continued reductions observed at 1- and 3-month follow-ups (Thorarinsdottir et al., 2021, 2022). Notably, in the current study a similar pattern of outcomes was achieved despite having fewer sessions and remote delivery of the intervention by non-specialists (i.e., individuals without professional training in mental health). Current data complement existing evidence of the efficacy of this intervention when delivered in acute settings in the initial hours and days following traumatic events (e.g., in women who experienced traumatic childbirth, Horsch et al., 2017; emergency department patients, (Iyadurai et al., 2018; Kanstrup, Singh, et al., 2021), and also in targeting established memories of trauma in frontline healthcare workers (Iyadurai et al., 2023; Ramineni et al., 2023).

We highlight that these findings are preliminary and come with several limitations, including a small sample size and (in keeping with the aims of a feasibility trial) the absence of a control condition. We note that intrusions increased from baseline to Week 1, and cannot rule out the possibility that the frequency of intrusive memories may have increased during the baseline phase due to participants being asked to monitor their occurrence, particularly in the context of long-standing trauma. This is an aspect that future studies may wish to carefully monitor and explore further – potentially through employing a larger sample size and extending the study period. Incorporating a longer baseline could also be beneficial to ascertain whether any observed increase is maintained or is transient. Further, this feasibility study cannot clarify the underlying mechanisms of the intervention; specifically, whether intrusions reduce owing to memory reconsolidation (Astill Wright et al., 2021), mental imagery interference (Baddeley & Andrade, 2000), a combination of the two, or other factors.

Should these beneficial outcomes be replicated and extended in randomised controlled trials, they will have important applied implications. Specifically, removing the need for in-person contact will increase the capacity to deliver the intervention at scale, and disseminate it to vulnerable traumatised populations (e.g., refugees), potentially overcoming challenges related to geographical location, language, and other barriers to access (Holmes et al., 2017; Kazlauskas, 2017). Similarly, eliminating the need for highly qualified trauma specialists to deliver the intervention competently will further increase scope for scalability.

## Supplementary Materials

The Supplementary Materials include the following items:

- the pre-registration protocol for the study, registered on ClinicalTrials.gov (NCT04709822) on 2021-01-14 (see Bjornsson, 2021)
- de-identified summary data, codebook and R scripts (see Gamble et al., 2022)
- online appendices (see Hardarson et al., 2024)

BjornssonA. S.
 (2021). Remote delivery of a brief visuospatial interference intervention to reduce intrusive memories of trauma
(ClinicalTrials.gov ID NCT04709822) [Pre-registration protocol]. PsychOpen. https://clinicaltrials.gov/study/NCT04709822


GambleB.
KanstrupM.
StephensenE. S.
BjornssonA. S.
HolmesE. A.
SinghL.
HardarsonJ. P.
ÞórarinsdóttirK.
MouldsM. L.
 (2022). Remote delivery of a brief visuospatial interference intervention to reduce intrusive memories among trauma exposed women: A feasibility study / De-identified data and analysis scripts
[De-identified summary data, codebook and R scripts]. PsychOpen. https://osf.io/2b6nh


HardarsonJ. P.
GambleB.
ThorarinsdottirK.
StephensenE. S.
KanstrupM.
GudmundssonT.
ValdimarsdóttirU.
HauksdottirA.
BjornssonA. S.
MouldsM. L.
HolmesE. A.
 (2024). Supplementary materials to "Developing a brief cognitive task intervention to reduce long-standing intrusive memories of trauma: A feasibility study with remote delivery for women in Iceland"
[Online appendices]. PsychOpen. 10.23668/psycharchives.14093
PMC1130391039119226

## Data Availability

Participants provided their consent for de-identified summary data to be made openly available for secondary research; this data can be accessed at the OSF (Gamble et al., 2022). We have aimed to follow FAIR data principles; i.e., such that data are findable, accessible, interoperable, and reusable. Study materials may be made available upon reasonable request with an appropriate materials transfer agreement (MTA) with EAH (Uppsala University). We note delivery of this intervention requires training and supervision.
